# Early Impact of VA MISSION Act Implementation on Primary Care Appointment Wait Time

**DOI:** 10.1007/s11606-022-07800-1

**Published:** 2022-10-28

**Authors:** Diana J. Govier, Alex Hickok, Samuel T. Edwards, Frances M. Weaver, Howard Gordon, Meike Niederhausen, Denise M. Hynes

**Affiliations:** 1 VA Portland Health Care System (VAPORHCS), Center to Improve Veteran Involvement in Care (CIVIC), Portland, OR USA; 2 OHSU - PSU School of Public Health, Oregon Health & Sciences University & Portland State University, Portland, OR USA; 3 Oregon Health & Sciences University, Portland, OR USA; 4 Edward Hines, Jr. VA Hospital, Center of Innovation for Complex Chronic Healthcare (CINCCH), Hines, IL USA; 5 Loyola University Chicago, Chicago, IL USA; 6 Jesse Brown VA Medical Center, Chicago, IL USA; 7 University of Illinois at Chicago, Chicago, IL USA; 8 College of Public Health and Human Sciences and the Center for Quantitative Life Sciences, Oregon State University, Corvallis, OR USA

**Keywords:** Veterans, primary, care, health, outcomes

## Abstract

**Background:**

Through Community Care Networks (CCNs) implemented with the VA MISSION Act, VA expanded provider contracting and instituted network adequacy standards for Veterans’ community care.

**Objective:**

To determine whether early CCN implementation impacted community primary care (PC) appointment wait times overall, and by rural/urban and PC shortage area (HPSA) status.

**Design:**

Using VA administrative data from February 2019 through February 2020 and a difference-in-differences approach, we compared wait times before and after CCN implementation for appointments scheduled by VA facilities that did (CCN appointments) and did not (comparison appointments) implement CCNs. We ran regression models with all appointments, and stratified by rural/urban and PC HPSA status. All models adjusted for Veteran characteristics and VA facility–level clustering.

**Appointments:**

13,720 CCN and 40,638 comparison appointments.

**Main Measures:**

Wait time, measured as number of days from authorization to use community PC to a Veteran’s first corresponding appointment.

**Key Results:**

Overall, unadjusted wait times increased by 35.7 days ([34.4, 37.1] 95% CI) after CCN implementation. In adjusted analysis, comparison wait times increased on average 33.7 days ([26.3, 41.2] 95% CI, *p* < 0.001) after CCN implementation; there was no significant difference for CCN wait times (across-group mean difference: 5.4 days, [−3.8, 14.6] 95% CI, *p* = 0.25). In stratified analyses, comparison wait time increases ranged from 29.6 days ([20.8, 38.4] 95% CI, *p* < 0.001) to 42.1 days ([32.9, 51.3] 95% CI, *p* > 0.001) after CCN implementation, while additional differences for CCN appointments ranged from 13.4 days ([3.5, 23.4] 95% CI, *p* = 0.008) to −15.1 days ([−30.1, −0.1] 95% CI, *p* = 0.05) for urban and PC HPSA appointments, respectively.

**Conclusions:**

After early CCN implementation, community PC wait times increased sharply at VA facilities that did and did not implement CCNs, regardless of rural/urban or PC HPSA status, suggesting community care demand likely overwhelmed VA resources such that CCNs had limited impact.

**Supplementary Information:**

The online version contains supplementary material available at 10.1007/s11606-022-07800-1.

## INTRODUCTION

Untimely healthcare can lead patients to seek care in emergency departments or forgo care altogether.^1^ The Veterans Health Administration (VA)―the largest US health system, with over 9 million enrolled Veterans^2^―is experiencing increased demand for care, which is contributing to longer wait times.^3–5^ In part in response to this issue, Congress passed the Veterans Access, Choice, and Accountability Act (Choice Act; Public Law 113-146) in 2014,^6^ which allowed Veterans to use VA-purchased care in the community if wait time, or travel distance/burden for VA facility care was too great. Yet, use of community primary care (PC) in the Choice program was initially low, in part due to limited numbers of community PC providers,^7, 8^ and wait time for community PC was significantly longer than at VA facilities during this time.^9^

To improve upon the Choice Act, in 2018 Congress passed the VA Maintaining Internal Systems and Strengthening Integrated Outside Networks Act (MISSION Act; Public Law 115-182),^10^ consolidating community care under the Veterans Community Care Program (VCCP) and broadening Veteran eligibility. Importantly, at a time when health plans are implementing narrow networks, steering patients to higher-value, lower-cost providers,^11^ the MISSION Act provisioned for new Community Care Networks (CCNs) comprising expanded networks of community providers, resulting in an increase of > 300,000 VA-contracted community providers (K. Mattocks, PhD, e-mail communication, November 2020). Furthermore, VA instituted new network adequacy standards—the ability to provide enrollees with timely access to an adequate number of in-network providers^12^—in CCNs^13^—the ability to provide enrollees with timely access to an adequate number of in-network providers. For PC, CCN network adequacy determinations are based on drive time of 30 to 60 min depending on level of rurality, and wait time of 30 days for routine appointments.^14^ Yet, like other health plans, VA has faced challenges setting and implementing these standards.^14^

Passage of the Choice and MISSION Acts has expanded VA’s role as purchaser of care. In 2021, VA allocated nearly one-quarter of its healthcare budget to purchasing community care and community care spending is estimated to reach $21.3 billion by 2022.^15^ Relatedly, research has documented increased demand for community care^16^ including PC^17^ among Veterans, portending new challenges and responsibilities for VA. For example, network adequacy determinations rely on the accuracy of provider directories, which are difficult to maintain and when inaccurate can lead to care delays and mask network deficiencies.^18, 19^ In addition, legislation mandates VA pay community providers Medicare rates, leaving VA with limited ability to regulate network adequacy through rate negotiations. Relatedly, VA facilities report strained relationships with community providers who refuse to accept VA patients.^16^ Compounding these issues, in rural areas where more Veterans live farther from VA facilities, initiatives to expand provider networks may be infeasible.^20^ Finally, because Veterans historically received most of their care at VA facilities, VA has had difficulty predicting demand for community care and targeting resources accordingly.^21^

To begin to assess the impact of the MISSION Act on Veteran care, we exploit a natural experiment of CCN contracting, studying whether wait time for community PC appointments changed after provider network expansions and new network adequacy standards. In addition, to explore the impact of CCNs in areas with more potential need for community care, we examine whether appointment wait times for Veterans residing in rural and PC health professional shortage areas changed after CCN implementation. Findings from this research will help purchasers including VA, providing evidence on whether larger provider networks coupled with network adequacy standards improves timeliness of care, or whether other strategies such as telehealth and subsidized transportation may be needed.

## METHODS

### Setting

VA has a unique community care appointment–making process (Appendix 1): First, a request or referral for community care is made.^21^ This is reviewed by VA staff and if eligible, the Veteran is authorized to use community care. Next, scheduling preferences are discussed with the Veteran, and VA or CCN staff schedule the appointment with a community provider. Authorizations for community care last from 3 to 12 months. If a Veteran has continued need for community care after an authorization ends, this process begins anew.

Prior to CCN implementation, community provider networks were administered by TriWest, a third-party administrator (TPA) who performed functions on behalf of VA such as scheduling appointments and paying community provider claims. With implementation of CCNs, VA now contracts with two TPAs, TriWest and Optum, which are tasked with developing and administering CCNs with transparency, accountability, quality, and in accordance with new network adequacy requirements. In addition, under CCNs, VA staff rather than TPAs are primarily responsible for scheduling community care appointments. Furthermore, VA modernized its information technology systems to reduce delays in community care eligibility determinations, appointments, and claims processing and payment.

The VA Office of Community Care developed a regional timeline for the transition from Choice-era to CCN contracts. VA facilities where CCNs are managed by Optum were the first to begin implementing CCN contacts (Appendix 2) between September and December 2019 in the Northeast; between November and December 2019 in the Midwest; and during November 2019 in the South. CCN regions managed by TriWest―the West and Alaska―were the last to implement CCN contracts in the latter half of 2020.

### Data and Study Population

We used data from the VA Corporate Data Warehouse (CDW),^22^ Health Resources and Services Administration (HRSA),^23^ and University of Wisconsin School of Medicine and Public Health.^24^ The CDW houses information from the VA electronic health record on Veteran characteristics, health conditions, and healthcare utilization. We linked information on Veteran address in the CDW with the University of Wisconsin Area Deprivation Index file and HRSA PC and mental health professional shortage area (HPSA) files. These data were linked with VA facility–level PC coordination scores from the VA Survey of Healthcare Experiences of Patients (SHEP).^25^

Our study population included community PC appointments that were authorized and took place between February 1, 2019, and February 29, 2020. To reduce potential bias related to process and outcome variation between the TriWest and Optum TPAs, appointments that took place in the West and Alaska CCN regions—regions managed by TriWest TPA where CCNs were not implemented until mid- to late-2020―were excluded from this analysis, which represented about half of community PC appointments during the study period. With these exclusions, the final study population comprised 54,358 community PC appointments among 32,002 Veterans (Appendix 3).

### Measures

#### Dependent Variable

Our dependent variable was wait time for first community PC appointment, measured as the number of days between a Veteran’s authorization for community PC and their first corresponding appointment (Steps 2 through 5, Appendix 1). If a Veteran had multiple authorizations during the study period, their first corresponding appointment after each authorization was included.

Following the approach outlined by Burgess et al. (2011), we identified PC appointments using procedure codes for PC and codes indicative of PC that could be accompanied by a PC visit (e.g., influenza immunizations, health screenings).^26^ To ensure appointments were for PC, we matched PC appointments to community PC authorization periods.

#### Independent Variables

Our main independent variables were a binary CCN appointment variable (CCN vs. comparison appointments [reference level]), a binary post-CCN implementation period variable (appointments that took place during vs. before CCN contracting [reference level]), and an interaction term for the CCN appointment and post-CCN period variables. A community care appointment was considered a CCN appointment if scheduled by a VA facility that implemented CCN contracting during the study period and a comparison appointment if scheduled by a VA facility that did not implement CCN contracting during the study period. For comparison appointments, the start of the post-CCN period was assigned as the median of the region-specific CCN contracting dates of sites that implemented CCN contracts.

#### Veteran-Level Covariates

Inclusion of Veteran-level covariates assigned to appointments was based on factors influencing Veteran healthcare use and appointment wait times.^17, 27–33^ Sociodemographic covariates included age group; sex; race; ethnicity; marital status; VA copay group; insurance coverage; and new VA enrollee status, where enrollees were considered new if they enrolled in VA after June 30, 2018. Regarding clinical covariates, we used the Elixhauser comorbidity method to calculate a risk-adjusted comorbidity score.^34, 35^ Geographic covariates included drive distance from Veteran residence to the nearest VA PC facility; rurality of residence; residence in a PC or mental health HPSA; and residence in a socioeconomically deprived area. Finally, we assigned VA facility–level PC coordination scores from the VA SHEP to Veterans’ appointments.^25^

### Statistical Analyses

Descriptive statistics included frequencies and percentages for categorical variables and means and SDs for continuous variables. To compare characteristics of CCN and comparison appointments, we calculated absolute standardized mean differences (SMD) between appointments.^36–38^

We employed a difference-in-differences (DID) approach and appointment-level multivariable linear regression models with VA facility–level clustering to estimate the effect of CCN contracting on wait times. We examined wait times for CCN and comparison appointments before and after CCN contracting and adjusted for covariates described above. In addition, we stratified analyses by rurality (rural and urban appointments) and PC HPSA status (PC HPSA and non-PC HPSA appointments).

All analyses were performed using R version 4.0.2.^39^ Cluster-robust standard errors were calculated using the multiwayvcov package version 1.2.3.^40^ For SMD, effect sizes were determined at 0.2 = small effect, 0.5 = medium effect, 0.8 = large effect.^41^ Statistical significance of regression model coefficients was determined at *p <* 0.05. The Hines and Portland VA Research Committees approved this project.

### Parallel Trends Analyses

**Figure 1:**
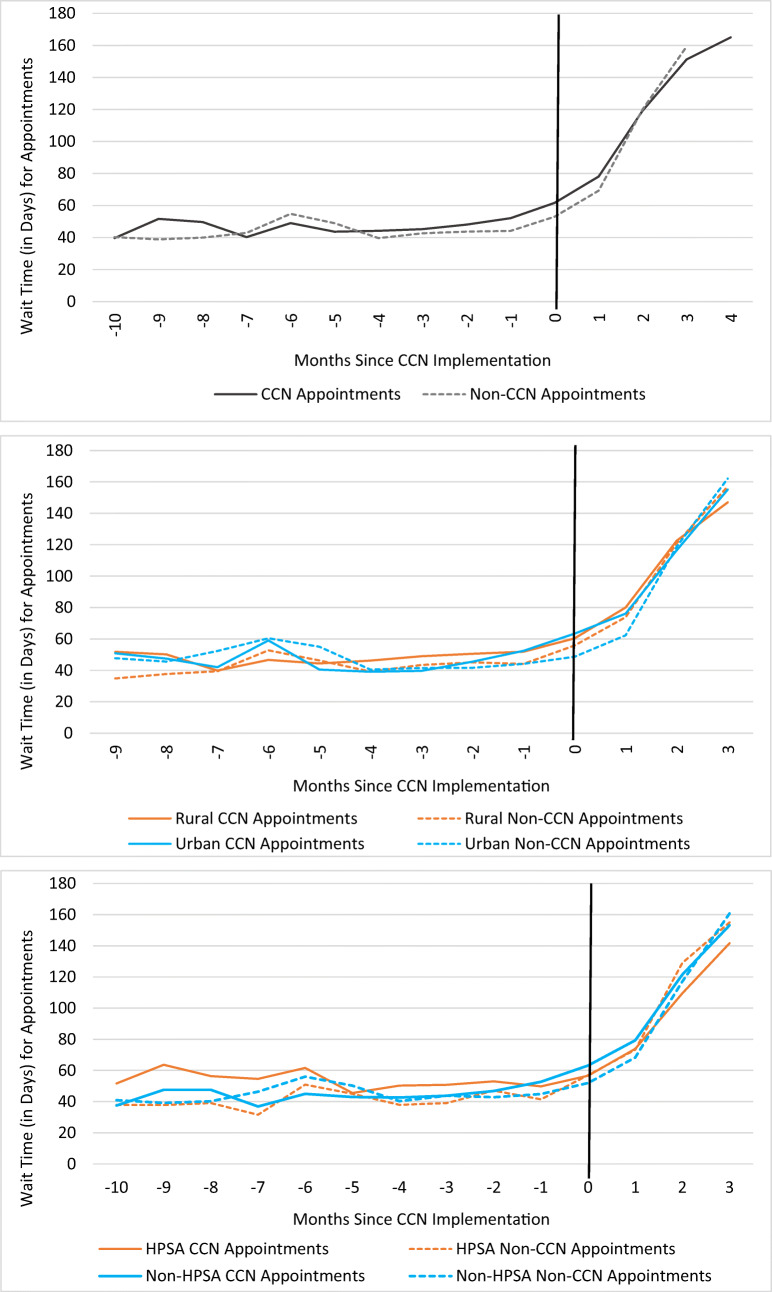
Unadjusted mean wait times for community primary care appointments by CCN status and months since CCN implementation (*N* = 54,358 appointments), overall and by rural/urban status and primary care HPSA status. Notes: The vertical line in each plot delineates the pre- and post-CCN periods. Due to the assignment of CCN contracting dates for comparison sites, comparison sites had a maximum of 3 months of post-CCN period data, whereas CCN sites had a maximum of 5 months of post-CCN period data. Months with unstable mean wait time estimates due to small group-specific cell size (*n* > 20 appointments) were excluded from plots. Abbreviations: CCN, Community Care Network; HPSA, health professional shortage area.

We examined the parallel trends assumption through examination of unadjusted wait times during the pre-CCN period (Fig. 1) and an appointment-level linear regression model similar to the main analysis but with a continuous month variable and an interaction term between the continuous month and CCN appointment variables. We found no evidence of differential trends in wait time between CCN and comparison appointments prior to CCN implementation (Appendix 4).

### Sensitivity Analyses

We conducted several sensitivity analyses for our overall model. First, we added Nosos score, a risk-adjustment measure that predicts healthcare costs for VA patients.^42^ Next, we tested the sensitivity of using the median for the assigned post-CCN period for comparison appointments, estimating models with alternative post-CCN periods. In four separate models, we assigned comparison appointments the same post period—September, October, November, or December. Finally, because some Veterans (50.2%) had multiple appointments, we tested a model that accounted for both VA facility–level and Veteran-level clustering.

## RESULTS

### Appointment Characteristics

During the study period, 54,358 community PC appointments took place (Table 1). Of those, about one-quarter were CCN appointments. CCN and comparison appointments were similar along most observable characteristics, and were primarily for Veterans who were older; male; white race; non-Hispanic/Latino ethnicity; married; and resided in rural areas and farther from VA PC facilities. Residence in socioeconomically deprived areas and mental health HPSAs occurred more often for comparison appointments, and VA facility–level SHEP care coordination scores were lower for comparison appointments.

**Table 1:** Community Primary Care  Appointment Characteristics by CCN Status (*N* = 54,358 appointments)

Characteristics	All	CCN	Comparison	Test statistic
	(*N* (%))	(*N* (%))	(*N* (%))	(SMD)
Unique appointments	54,358 (100)	13,720 (25.2)	40,638 (74.8)	
Unique patients	32,002 (100)	8477 (26.5)	23,525 (73.5)	
*Age category*				0.04
< 45 years	14,030 (25.8)	3624 (26.4)	10,406 (25.6)	
45–54 years	8779 (16.2)	2307 (16.8)	6472 (15.9)	
55–64 years	12,396 (22.8)	3152 (23.0)	9244 (22.7)	
≥ 65 years	19,153 (35.2)	4637 (33.8)	14,516 (35.7)	
Sex: female	6335 (11.7)	1699 (12.4)	4636 (11.4)	0.03
*Race*				0.18
White	43,027 (79.2)	10,313 (75.2)	32,714 (80.5)	
Black	8131 (15.0)	2722 (19.8)	5409 (13.3)	
Other	3200 (5.9)	685 (5.0)	2515 (6.2)	
Ethnicity: Hispanic/Latino	4654 (8.6)	464 (3.4)	4190 (10.3)	0.28
Marital status: single/unknown	23,932 (44.0)	6422 (46.8)	17,510 (43.1)	0.09
*VA copayment category*				0.09
No copay	31,750 (58.4)	8474 (61.8)	23,276 (57.3)	
Some copay	16,458 (30.3)	3802 (27.7)	12,656 (31.1)	
Full copay	6150 (11.3)	1444 (10.5)	4706 (11.6)	
New VA enrollee	5125 (9.4)	1829 (13.3)	3296 (8.1)	0.17
*Insurance status*				0.15
VA only	19,518 (35.9)	5442 (39.7)	14,076 (34.6)	
VA + private	9674 (17.8)	2161 (15.8)	7513 (18.5)	
VA + Medicare/Medicaid	16,331 (30.0)	3637 (26.5)	12,694 (31.2)	
VA + private + Medicare/Medicaid	8835 (16.3)	2480 (18.1)	6355 (15.6)	
Elixhauser score (mean (SD))	6.79 (11.96)	6.69 (12.00)	6.83 (11.95)	0.01
Facility-level SHEP primary care coordination score	60.19 (6.15)	62.99 (6.67)	59.24 (5.66)	0.61
County Average National ADI Rank (mean (SD))	67.89 (14.76)	61.15 (17.96)	70.16 (12.72)	0.58
Primary care HPSA	12,359 (22.7)	2930 (21.4)	9429 (23.2)	0.04
Mental health HPSA	32,480 (59.8)	5359 (39.1)	27,121 (66.7)	0.58
*Rurality of residence*				0.07
Urban	19,464 (35.8)	5266 (38.4)	14,198 (34.9)	
Rural	31,548 (58.0)	7622 (55.6)	23,926 (58.9)	
Highly rural	3346 (6.2)	832 (6.1)	2514 (6.2)	
*Distance to nearest VA primary care facility*				0.15
0–5 miles	5692 (10.5)	1497 (10.9)	4195 (10.3)	
6–10 miles	6473 (11.9)	1744 (12.7)	4729 (11.6)	
11–20 miles	8113 (14.9)	2313 (16.9)	5800 (14.3)	
21–40 miles	12,044 (22.2)	3326 (24.2)	8718 (21.5)	
> 40 miles	22,036 (40.5)	4840 (35.3)	17,196 (42.3)	

Abbreviations: *CCN*, Community Care Network; *SMD*, standardized mean differences; *VA*, Veterans Affairs; *ADI*, Area Deprivation Index; *HPSA*, health professional shortage area; *SHEP*, Survey of Healthcare Experiences of Patients

### Wait Times

Wait times for community PC appointments were skewed right with mean 52.2 days and median 30.0 days (data not shown), averaging 44.4 days (95% CI 43.8, 44.9) and 80.1 days (95% CI 78.9, 81.4) during the pre- and post-CCN periods, respectively (Table 2, Fig. 1). The unadjusted mean wait time for a CCN appointment was longer than for a comparison appointment in both the pre- and post-periods.

**Table 2:** Wait Times for Community Primary Care Appointments, Pre- and Post-CCN Implementation

	All	CCN	Comparison
	*N* = 54,358	*N* = 13,720	*N* = 40,638
*Unadjusted (mean/95% CI)*			
Pre-CCN	44.4 (43.8, 44.9)	47.4 (46.4, 48.4)	43.4 (42.8, 44.1)
Post-CCN	80.1 (78.9, 81.4)	86.8 (84.7, 88.9)	76.9 (75.4, 78.5)
Within-group difference	35.7 (34.4, 37.1)	39.4 (37.1, 41.8)	33.5 (31.8, 35.1)
Across-group difference		6.0 (3.3, 8.6)	
*Adjusted (mean/95% CI)*			
Pre-CCN	―	44.6 (36.6, 52.6)	45.6 (36.3, 54.9)
Post-CCN	―	83.8 (75.5, 92.0)	79.3 (68.2, 90.4)
Within-group difference	―	39.2 (33.5, 44.8)	33.7 (26.3, 41.2)
Across-group difference		5.4 (−3.8, 14.6)	

Notes: Adjusted means and 95% CIs are from multivariable linear regression models that adjusted for VA facility–level clustering and age category, sex, race, ethnicity, marital status, VA copayment category, new VA enrollee status, insurance status, county-level national ADI rank of Veteran residence, Veteran residence in a primary care HPSA, Veteran residence in a mental health HPSA, rurality of Veteran residence, distance to the nearest VA primary care facility from Veteran residence, VA facility–level SHEP care coordination score, and Elixhauser index

Abbreviations: *CCN*, Community Care Network; *ADI*, Area Deprivation Index; *HPSA*, health professional shortage area; *SHEP*, Survey of Healthcare Experiences of Patients

In the adjusted regression analysis, comparison appointment wait times increased on average 33.7 days ([26.3, 41.2] 95% CI, *p* < 0.001) after CCN implementation; there was no significant difference between CCN and comparison appointment wait times after CCN implementation (across-group mean difference: 5.4 days, [−3.82, 14.64] 95% CI, *p* = 0.25; Table 2; Appendix 5). In sensitivity analyses, altering our model parameters and assumptions did not materially impact these results (Appendix 6).

#### Wait Times by Rural/Urban Status and Primary Care HPSA Status

In the adjusted stratified analyses, rural comparison wait times increased 36.6 days ([29.6, 43.6] 95% CI, *p* < 0.001) after CCN implementation; there was no significant difference between rural CCN and comparison appointment wait times after CCN implementation (across-group mean difference: 0.4 days, [−10.8, 11.5] 95% CI, *p* = 0.95). Urban comparison wait times increased 29.6 days ([20.8, 38.4] 95% CI, *p* < 0.001), and urban CCN wait times an additional 13.4 days ([3.5, 23.4] 95% CI, *p* = 0.008; Table 3; Appendix 7) after CCN implementation.

**Table 3:** Wait Time for Community Primary Care Appointments, Pre- and Post-CCN Implementation, by Rural/Urban Status

	Rural appointments			Urban appointments		
	All	CCN	Comparison	All	CCN	Comparison
	*N* = 34,894	*N* = 8454	*N* = 26,440	*N* = 19,464	*N* = 5266	*N* = 14,198
*Unadjusted (mean/95% CI)*						
Pre-CCN	43.7 (43.0, 44.3)	48.1 (46.8, 49.4)	42.3 (41.6, 43.1)	45.7 (44.9, 46.6)	46.0 (44.6, 47.4)	45.6 (44.6, 46.7)
Post-CCN	80.8 (79.1, 82.5)	85.3 (82.1, 88.5)	78.9 (76.9, 80.9)	79.2 (77.4, 81.1)	88.4 (85.6, 91.2)	73.9 (71.5, 76.3)
Within-group difference	37.1 (35.3, 38.9)	37.2 (33.8, 40.7)	36.6 (34.4, 38.7)	33.5 (31.4, 35.5)	42.4 (39.2, 45.5)	28.3 (25.7, 30.9)
Across-group difference		0.7 (−2.9, 4.2)			14.1 (10.2, 18.0)	
*Adjusted (mean/95% CI)*						
Pre-CCN	―	47.5 (39.6, 55.3)	45.2 (37.6, 52.9)	―	43.3 (35.1, 51.6)	52.4 (42.9, 61.8)
Post-CCN	―	84.5 (73.2, 95.7)	81.8 (72.9, 90.6)	―	86.3 (78.4, 94.3)	81.9 (70.3, 93.6)
Within-group difference	―	37.0 (28.3, 45.7)	36.6 (29.6, 43.6)	―	43.0 (38.5, 47.5)	29.6 (20.8, 38.4)
Across-group difference		0.4 (−10.8, 11.5)			13.4 (3.5, 23.4)	

Notes: Adjusted means and 95% CIs are from multivariable linear regression models that adjusted for VA facility–level clustering and age category, sex, race, ethnicity, marital status, VA copayment category, new VA enrollee status, insurance status, county-level national ADI rank of Veteran residence, Veteran residence in a primary care HPSA, Veteran residence in a mental health HPSA, distance to the nearest VA primary care facility from Veteran residence, VA facility–level SHEP care coordination score, and Elixhauser index

Abbreviations: *CCN*, Community Care Network; *ADI*, Area Deprivation Index; *HPSA*, health professional shortage area; *SHEP*, Survey of Healthcare Experiences of Patients

PC HPSA comparison wait times increased 42.1 days ([32.9, 51.3] 95% CI, *p* < 0.001) after CCN implementation, and PC HPSA CCN wait times a lesser 15.1 days ([−30.1, −0.1] 95% CI, *p* = 0.049); non-PC HPSA comparison wait times increased 31.5 days ([23.9, 39.0] 95% CI, *p* < 0.001), and non-PC HPSA CCN wait times an additional 11.0 days ([2.2, 19.8] 95% CI, *p* = 0.014; Table 4; Appendix 8) after CCN implementation.

**Table 4:** Wait Time for Community Primary Care Appointments, Pre- and Post-CCN Implementation, by Primary Care HPSA Status

	Primary care HPSA			Non-primary care HPSA		
	All	CCN	Comparison	All	CCN	Comparison
	*N* = 12,359	*N* = 2930	*N* = 12,359	*N* = 41,999	*N* = 10,790	*N* = 31,209
*Unadjusted (mean/95% CI)*						
Pre-CCN	43.4 (42.3, 44.5)	52.6 (50.1, 55.1)	40.9 (39.6, 42.2)	44.6 (44.0, 45.2)	46.0 (44.9, 47.0)	44.2 (43.5, 44.9)
Post-CCN	81.7 (78.9, 84.4)	79.3 (74.7, 84.0)	82.8 (79.4, 86.2)	79.7 (78.3, 81.1)	88.9 (86.5, 91.3)	75.3 (73.6, 77.0)
Within-group difference	38.3 (35.3, 41.2)	26.8 (21.5, 32.0)	41.9 (38.2, 45.5)	35.0 (33.5, 36.6)	42.9 (40.3, 45.5)	31.1 (29.2, 32.9)
Across-group difference		−15.1 (−20.9, −9.3)			11.8 (8.9, 14.8)	
*Adjusted (mean/95% CI)*						
Pre-CCN	―	48.3 (35.8, 60.8)	39.6 (29.2, 49.9)	―	43.8 (35.8, 51.8)	47.5 (37.4, 57.6)
Post-CCN	―	75.4 (60.8, 89.9)	81.7 (66.0, 97.4)	―	86.3 (78.6, 94.0)	78.9 (67.6, 90.2)
Within-group difference	―	27.0 (15.4, 38.6)	42.1 (32.9, 51.3)	―	42.5 (37.5, 47.4)	31.5 (23.9, 39.0)
Across-group difference		−15.1 (−30.1, −0.1)			11.0 (2.2, 19.8)	

Notes: Adjusted means and 95% CIs are from multivariable linear regression models that adjusted for VA facility–level clustering and age category, sex, race, ethnicity, marital status, VA copayment category, new VA enrollee status, insurance status, county-level national ADI rank of Veteran residence, Veteran residence in a mental health HPSA, rurality of Veteran residence, distance to the nearest VA primary care facility from Veteran residence, VA facility–level SHEP care coordination score, and Elixhauser index

Abbreviations: *CCN*, Community Care Network; *ADI*, Area Deprivation Index; *HPSA*, health professional shortage area; *SHEP*, Survey of Healthcare Experiences of Patients

## DISCUSSION

This study is among the first to examine the impact of the MISSION Act, assessing wait time for community PC appointments after early CCN implementation. We found that early CCN implementation was not associated with timelier community PC during the study period. Instead, wait times increased significantly and similarly for CCN and comparison appointments after CCN implementation. In addition, although there were some differences in the magnitude of wait time increases stratified by rural/urban and PC HPSA status, wait times increased sharply for both CCN and comparison appointments after CCN implementation, ranging from approximately 30 to 40 days, which is beyond VA’s new network adequacy wait time standard for community care.

Our results are consistent with a recent Government Accountability Office (GAO) report, which showed that in some areas VA underestimated demand for community care leading to delays.^21^ Initially, VA assumed a 10% growth in community care referrals with VCCP implementation. However, referral growth at some VA facilities ranged from 40 to 70% between June 2019 and February 2020. Lacking accurate estimates of demand, staffing models were impacted resulting in inadequate staffing to meet even modest referral growth projections. In addition, that wait time increases were somewhat smaller for rural versus urban CCN appointments and PC HPSA versus non-PC HPSA CCN appointments—areas where fewer providers may be available for CCN expansion, but where VA may have more experience managing greater community care referral volume—is also consistent with the GAO report and suggest VA’s in/ability to manage community care demand likely contributed to our results.

Next, it is plausible that even with expanded provider networks, the number of providers furnishing VA community care may not have increased substantially and CCN directories may include inactive providers, leading to challenges scheduling appointments. This phenomenon has been observed in Medicaid managed care (MCO) networks.^43, 44^ For example, 25% of PC providers in Medicaid MCOs provided 86% of enrollee care from 2015 to 2017;^43^ and in 2018 more than half of some Medicaid MCO PC provider networks were comprised of “phantom” providers who had not seen Medicaid enrollees.^44^ The extent to which VA network adequacy standards may not reflect actual access to care warrants further investigation, as well as dissemination of lessons learned that may inform other public and commercial health plans.

Finally, given specialty care historically comprised most VA community care,^16^ priority may have been given to increasing specialist rather than PC networks. Coupled with expanded Veteran eligibility for community care, this focus on CCN specialist care may have exacerbated supply-demand disequilibrium for community PC. Future research should explore which provider types were targeted for CCN expansion and the extent to which expansion aligned with changing demand for community care, including PC.

Given the myriad consequences of delayed access to PC, including deferred care, emergency department and hospital use, and poor health outcomes,^27–31^ it is critical that VA carefully consider whether and when to send Veterans out for community PC. VA leaders and policymakers recognize the importance of these decisions and are developing solutions, such as transferring referral management from individual clinicians to referral teams trained to optimize provider and setting selection.^45^ One pilot study testing this approach for sleep medicine found VA wait times were shorter under the referral team than status quo, although testing for PC has not yet been done.^45^ Alternatively, proponents have suggested strategic community provider contracting based on provider performance—a strategy akin to narrow network contracting in which provider networks are curated based on quality and costs,^11^ one VA is currently barred from practicing.^46^ Each strategy assumes a different underlying mechanism for wait times, yet, each requires accurate, real-time data on community provider availability, performance, and willingness to care for Veterans.

### Limitations

This study has some limitations. First, to isolate the impact of CCNs from other VA appointment–making processes that may contribute to wait times, we explored wait time as the difference between the date an appointment was scheduled and when it took place (Steps 4 through 5, Appendix 1). However, appointment scheduled dates were missing during certain periods of our study. Therefore, our measure of wait time includes both wait time attributable to CCN and other VA appointment–making factors, and does not include cancelled appointments. Although cancellations account for a small percentage of VA appointments (2.3%),^47^ once data are available, future research should explore the impact of CCNs on cancelled appointments. In addition, we did not have information on appointment desired dates, and as such, were unable to determine whether wait times were planned. By employing a DID approach, however, unless CCN and comparison appointments were systematically different in their scheduled or desired dates, potential bias should be minimal. Lastly, there were 5 months of post-CCN data before the COVID-19 pandemic began disrupting care. Therefore, we were unable to assess the longer-range impact of CCNs on wait times. After the pandemic subsides, it will be important to monitor community care wait times and the impact of additional experience with CCNs and new network adequacy standards.

## CONCLUSION

Contrary to what architects of the MISSION Act intended, findings from our study indicate that expanded contracting with community providers and new provider network adequacy standards implemented through CCNs did not, in early stages, improve timeliness of community PC among Veterans. To develop targeted solutions, research is needed to disentangle the potential impacts of staff and process issues from community provider network issues and adequacy standards on appointment wait times. Insights from these inquiries may inform, more broadly, approaches to better estimate and manage demand for services and wait times in health plan networks.

## Supplementary Information

ESM 1 (DOCX 251 kb)

## Data Availability

The datasets generated and analyzed during the current study are not publicly available due to human subjects protection requirements. Requests for summary information may be submitted to the corresponding author for consideration.
