# Supplementary material for: Early Impact of VA MISSION Act Implementation on Primary Care Appointment Wait Time
Source: J Gen Intern Med. 2022 Oct 28;38(4):889–97. doi: 10.1007/s11606-022-07800-1 (PMC9616400; doi:10.1007/s11606-022-07800-1)
Supplement: Supplementary file 1 — (DOCX 251 kb) [file 11606_2022_7800_MOESM1_ESM.docx]

APPENDICES

Appendix 1. Overview of the VA Community Care Appointment-Making Process.

Abbreviations: CCN, Community Care Network.

Appendix 2. Map of CCN Regions and VA Facilities


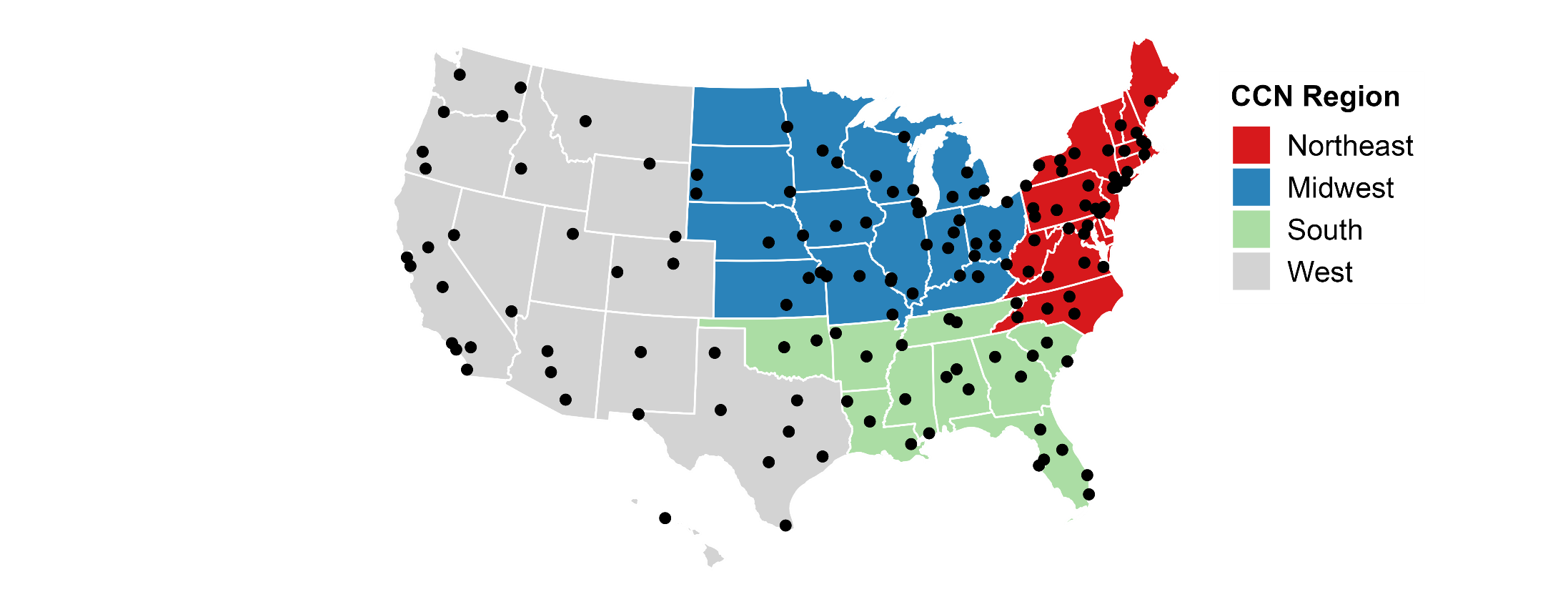


Notes: Dots represent VA facilities. Alaska (CCN Region 5) and U.S territories not shown. West (CCN Region 4) excluded from analyses. Abbreviations: CCN, Community Care Network.

Appendix 3. Study Population Flow Diagram

Community primary care appointments between February 1, 2019 – February 29, 2020

(n= 64,830 Veterans / 113,900 appointments)

Excluded: Outside of contiguous U.S. or in West Community Care Network region [n=31,847 (49.1%) Veterans / 57,862 (50.8%) appointments]

Comparison appointments (n=24,240 Veterans / 41,847 appointments): Community primary care appointments that took place between February 1, 2019 – February 29, 2020, that were scheduled by VA facilities that had not yet implemented Community Care Network contracts

CCN appointments (n=8,772 Veterans / 14,191 appointments): Community primary care appointments that took place between February 1, 2019 – February 29, 2020, that were scheduled by VA facilities that implemented Community Care Network contracts

Missing covariate information [n=295 (3.3%) Veterans / 471 (3.3%) appointments]

Analysed (n=8,477 Veterans / 13,720 appointments)

Missing covariate information [n=715 (2.9%) Veterans / 1,209 (2.9%) appointments]

Analysed (n=23,525 Veterans / 40,638 appointments)

Appendix 4. Test of Parallel Trends in Wait Times for Appointments During the Pre-CCN Period

|  | Coefficient (Days) (95% CI) | 95% Confidence Interval | P-Value |
| --- | --- | --- | --- |
| CCN appointment | -2.49 | (-43.78, 38.80) | 0.91 |
| Study period month | 0.30 | (-0.64, 1.23) | 0.53 |
| CCN appointment X study period month | 0.05 | (-1.26, 1.36) | 0.94 |
| *Age category (reference <45 years)* |  |  |  |
| 45-54 years | 0.72 | (-1.39, 2.83) | 0.50 |
| 55-64 years | -0.19 | (-2.38, 2.01) | 0.87 |
| ≥65 years | 1.16 | (-1.19, 3.51) | 0.33 |
| *Sex (reference Male)* |  |  |  |
| Female | -2.88 | (-5.31, -0.45) | 0.02 |
| *Race (reference White)* |  |  |  |
| Black | 5.65 | (2.04, 9.27) | 0.002 |
| Other/Unknown | 2.08 | (-0.43, 4.59) | 0.10 |
| *Ethnicity (reference Non-Hispanic/Latino)* |  |  |  |
| Hispanic/Latino | 3.70 | (-7.24, -0.16) | 0.04 |
| *Marital status (reference Married)* |  |  |  |
| Single/Other | 0.77 | (-0.46, 2.01) | 0.22 |
| *VA copayment category (reference No copay)* |  |  |  |
| Some copay | 0.56 | (-0.75, 1.87) | 0.41 |
| Full copay | 3.80 | (1.33, 6.27) | 0.003 |
| New VA enrollee | -1.72 | (-4.13, 0.68) | 0.16 |
| *Insurance status (reference VA only)* |  |  |  |
| VA + private | -0.58 | (-2.20, 1.04) | 0.49 |
| VA + Medicare/Medicaid | 1.44 | (-1.51, 4.40) | 0.34 |
| VA, + private, + Medicare/Medicaid | 4.15 | (1.69, 6.61) | 0.001 |
| Elixhauser score, 10 points | -0.67 | (-1.51, 0.18) | 0.12 |
| VA facility-level SHEP care coordination score | 0.50 | (0.16, 0.83) | 0.004 |
| County-Level National ADI Rank | -0.11 | (-0.24, 0.02) | 0.10 |
| Primary care HPSA | 1.10 | (-2.44, 4.65) | 0.54 |
| Mental health HPSA | -5.23 | (-9.65, -0.82) | 0.02 |
| *Rurality of residence (reference Urban)* |  |  |  |
| Rural | 0.78 | (-3.53, 5.10) | 0.72 |
| Highly rural | 0.14 | (-6.08, 6.36) | 0.97 |
| *Distance to nearest VA primary care facility (reference 0-5 miles)* |  |  |  |
| 6-10 miles | 0.60 | (-2.87, 4.07) | 0.74 |
| 11-20 miles | -1.25 | (-5.47, 2.97) | 0.56 |
| 21-40 miles | -3.8 | (-9.28, 1.69) | 0.18 |
| >40 miles | -3.57 | (-9.53, 2.38) | 0.24 |

Notes: Coefficients, 95% confidence intervals, and p-values are from an appointment-level multivariable linear regression model with VA facility-level cluster-robust standard errors.

Abbreviations: VA, Veterans Affairs; CCN, Community Care Networks; ADI, Area Deprivation Index; HPSA, health professional shortage area; SHEP, Survey of Healthcare Experiences of Patients.

Appendix 5. Full Regression Results for the Effect of CCN Contracting on Wait Time for Appointments (N=54,358 appointments)

|  | Coefficient (Days) | 95% Confidence Interval | P-Value |
| --- | --- | --- | --- |
| CCN appointment | -0.96 | (-6.14, 4.21) | 0.72 |
| Post-CCN period | 33.74 | (26.30, 41.18) | <0.001 |
| CCN appointment x post-CCN period | 5.41 | (-3.82, 14.64) | 0.25 |
| *Age category (reference <45 years)* |  |  |  |
| 45-54 years | -0.16 | (-2.39, 2.07) | 0.89 |
| 55-64 years | -0.73 | (-3.29, 1.82) | 0.58 |
| ≥65 years | 1.06 | (-1.49, 3.61) | 0.42 |
| *Sex (reference Male)* |  |  |  |
| Female | -2.04 | (-4.40, 0.32) | 0.09 |
| *Race (reference White)* |  |  |  |
| Black | 5.19 | (2.01, 8.37) | 0.001 |
| Other/Unknown | 1.78 | (-0.71, 4.26) | 0.16 |
| *Ethnicity (reference Non-Hispanic/Latino)* |  |  |  |
| Hispanic/Latino | -3.64 | (-7.84, 0.56) | 0.09 |
| *Marital status (reference Married)* |  |  |  |
| Single/Other | 1.47 | (0.33, 2.61) | 0.01 |
| *VA copayment category (reference No copay)* |  |  |  |
| Some copay | 0.37 | (-0.90, 1.64) | 0.57 |
| Full copay | 3.64 | (1.46, 5.82) | 0.001 |
| New VA enrollee | -0.63 | (-3.17, 1.91) | 0.63 |
| *Insurance status (reference VA only)* |  |  |  |
| VA + private | 0.07 | (-1.55, 1.68) | 0.94 |
| VA + Medicare/Medicaid | 2.66 | (0.00, 5.33) | 0.05 |
| VA, + private, + Medicare/Medicaid | 6.02 | (3.36, 8.69) | <0.001 |
| Elixhauser score, 10 points | -0.75 | (-1.44, -0.05) | 0.04 |
| VA facility-level SHEP care coordination score | 0.55 | (0.24, 0.86) | 0.001 |
| County-Level National ADI Rank | -0.12 | (-0.24, 0.01) | 0.07 |
| Primary care HPSA | 1.57 | (-1.43, 4.58) | 0.31 |
| Mental health HPSA | -4.80 | (-9.53, -0.07) | 0.05 |
| *Rurality of residence (reference Urban)* |  |  |  |
| Rural | 0.70 | (-3.97, 5.37) | 0.77 |
| Highly rural | -0.13 | (-6.86, 6.59) | 0.97 |
| *Distance to nearest VA primary care facility (reference 0-5 miles)* |  |  |  |
| 6-10 miles | 0.60 | (-2.88, 4.08) | 0.74 |
| 11-20 miles | -1.19 | (-5.13, 2.76) | 0.56 |
| 21-40 miles | -3.29 | (-8.58, 1.99) | 0.22 |
| >40 miles | -2.62 | (-8.87, 3.63) | 0.41 |

Notes: Coefficients, 95% confidence intervals, and p-values are from an appointment-level multivariable linear regression model with VA facility-level cluster-robust standard errors.

Abbreviations: VA, Veterans Affairs; CCN, Community Care Networks; ADI, Area Deprivation Index; HPSA, Health Professional Shortage Area; SHEP, Survey of Healthcare Experiences of Patients.

Appendix 6. Comparison of Main Results for Primary and Sensitivity Analysis Models

|  | Coefficient (Days) | | | 95% Confidence Interval | | | P-Value |
| --- | --- | --- | --- | --- | --- | --- | --- |
| *Primary Model* |  | | |  | | |  |
| CCN appointment | -0.96 | | | (-6.14, 4.21) | | | 0.72 |
| Post-CCN period | 33.74 | | | (26.30, 41.18) | | | <0.001 |
| CCN appointment x post-CCN period | 5.41 | | | (-3.82, 14.64) | | | 0.25 |
| *With Veteran and Facility-Level Clustering* | | |  |  | | |  |
| CCN appointment | -0.96 | | | (-6.14, 4.22) | | | 0.72 |
| Post-CCN period | 33.74 | | | (26.30, 41.18) | | | <0.001 |
| CCN appointment x post-CCN period | 5.41 | | | (-3.82, 14.65) | | | 0.25 |
| *With Nosos Score* |  | | |  | | |  |
| CCN appointment | -1.47 | | | (-6.79, 3.85) | | | 0.59 |
| Post-CCN period | 34.89 | | | (27.89, 41.89) | | | <0.001 |
| CCN appointment x post-CCN period | 5.6 | | | (-3.54, 14.73) | | | 0.23 |
| Nosos Score | -1.05 | | | (-1.52, -0.59) | | | <0.001 |
| *With September Imputed Post Period* |  |  | | |  |  |  |
| CCN appointment | -0.79 | | | (-7.44, 5.86) | | | 0.82 |
| Post-CCN period | 14.99 | | | (9.70, 20.27) | | | <0.001 |
| CCN appointment x post-CCN period | 6.79 | | | (-1.13, 14.70) | | | 0.09 |
| *With October Imputed Post Period* |  |  | | |  |  |  |
| CCN appointment | -0.83 | | | (-6.91, 5.26) | | | 0.79 |
| Post-CCN period | 20.02 | | | (14.89, 21.14) | | | <0.001 |
| CCN appointment x post-CCN period | 8.03 | | | (-0.15, 15.90) | | | 0.05 |
| *With November Imputed Post Period* |  |  | | |  |  |  |
| CCN appointment | -0.28 | | | (-5.71, 5.14) | | | 0.92 |
| Post-CCN period | 31.01 | | | (24.86, 37.16) | | | <0.001 |
| CCN appointment x post-CCN period | 7.72 | | | (-0.50, 15.95) | | | 0.07 |
| *With December Imputed Post Period* |  |  | | |  |  |  |
| CCN appointment | 0.42 | | | (-4.63, 5.48) | | | 0.87 |
| Post-CCN period | 48.5 | | | (41.36, 55.65) | | | <0.001 |
| CCN appointment x post-CCN period | 2.76 | | | (-6.24, 11.75) | | | 0.55 |

Notes: Coefficients, 95% confidence intervals, and p-values are from appointment-level multivariable linear regression models with VA facility-level cluster-robust standard errors, except where stated otherwise. Covariates in models are identical to those in the primary model except where stated otherwise.

Abbreviations: CCN, Community Care Network.

Appendix 7. Full Regression Results for the Effect of CCN Contracting on Wait Time for Appointments, Stratified by Rural/Urban Status

|  | Rural Appointments (N=34,894) | | Urban Appointments (N=19,464) | |
| --- | --- | --- | --- | --- |
|  | Coefficient (Days) | 95% Confidence Interval | Coefficient (Days) | 95% Confidence Interval |
| CCN appointment | 2.32 | (-2.55, 7.20) | -9.01 | (-17.36, -0.66) |
| Post-CCN period | 36.59 | (29.55, 43.64) | 29.58 | (20.80, 38.36) |
| CCN appointment x post-CCN period | 0.39 | (-10.77, 11.54) | 13.42 | (3.45, 23.39) |
| *Age category (reference <45 years)* |  |  |  |  |
| 45-54 years | -0.88 | (-3.59, 1.83) | -0.09 | (-3.25, 3.08) |
| 55-64 years | -2.17 | (-4.78, 0.44) | 0.32 | (-4.11, 4.75) |
| ≥65 years | -0.01 | (-2.82, 2.81) | 1.81 | (-3.18, 6.80) |
| *Sex (reference Male)* |  |  |  |  |
| Female | -2.26 | (-5.14, 0.62) | -1.36 | (-4.76, 2.05) |
| *Race (reference White)* |  |  |  |  |
| Black | 6.93 | (3.91, 9.95) | 3.07 | (-0.47, 6.61) |
| Other/Unknown | 2.20 | (-1.91, 6.32) | 2.04 | (-0.87, 4.96) |
| *Ethnicity (reference Non-Hispanic/Latino)* |  |  |  |  |
| Hispanic/Latino | -2.05 | (-5.84, 1.74) | -3.07 | (-7.47, 1.33) |
| *Marital status (reference Married)* |  |  |  |  |
| Single/Other | 1.03 | (-0.65, 2.70) | 1.69 | (0.39, 2.98) |
| *VA copayment category (reference No copay)* |  |  |  |  |
| Some copay | 0.29 | (-1.11, 1.69) | 0.53 | (-1.91, 2.97) |
| Full copay | 3.76 | (1.23, 6.30) | 2.65 | (-0.46, 5.76) |
| New VA enrollee | -0.48 | (-3.74, 2.77) | -0.43 | (-3.71, 2.85) |
| *Insurance status (reference VA only)* |  |  |  |  |
| VA + private | -0.81 | (-2.68, 1.06) | 2.98 | (-1.50, 7.46) |
| VA + Medicare/Medicaid | 3.45 | (0.66, 6.23) | 2.48 | (-0.51, 5.47) |
| VA, + private, + Medicare/Medicaid | 5.84 | (2.76, 8.92) | 6.02 | (1.43, 10.60) |
| Elixhauser score, 10 points | -1.00 | (-1.70, -0.29) | -0.47 | (-1.48, 0.54) |
| VA facility-level SHEP care coordination score | 0.45 | (0.12, 0.77) | 0.64 | (0.17, 1.12) |
| County-Level National ADI Rank | -0.11 | (-0.22, 0.00) | -0.09 | (-0.25, 0.08) |
| Primary care HPSA | 0.94 | (-1.57, 3.45) | 3.78 | (-3.29, 10.85) |
| Mental health HPSA | -0.56 | (-4.44, 3.31) | -14.22 | (-21.88, -6.57) |
| *Distance to nearest VA primary care facility (reference 0-5 miles)* |  |  |  |  |
| 6-10 miles | -2.86 | (-9.33, 3.61) | 1.26 | (-2.19, 4.72) |
| 11-20 miles | -3.24 | (-10.65, 4.16) | -1.54 | (-5.95, 2.87) |
| 21-40 miles | -4.27 | (-11.26, 2.72) | -6.63 | (-12.68, -0.57) |
| >40 miles | -4.22 | (-10.96, 2.52) | -3.23 | (-10.74, 4.27) |

Notes: Coefficients and 95% confidence intervals are from appointment-level multivariable linear regression models with VA facility-level cluster-robust standard errors.

Abbreviations: VA, Veterans Affairs; CCN, Community Care Networks; ADI, Area Deprivation Index; HPSA, Health Professional Shortage Area; SHEP, Survey of Healthcare Experiences of Patients.

Appendix 8. Full Regression Results for the Effect of CCN Contracting on Wait Time for Appointments, Stratified by Primary Care HPSA Status

|  | HPSA Appointments (N=12,359) | | Non-HPSA Appointments (N=41,999) | |
| --- | --- | --- | --- | --- |
|  | Coefficient (Days) | 95% Confidence Interval | Coefficient (Days) | 95% Confidence Interval |
| CCN appointment | 8.78 | (1.80, 15.75) | -3.64 | (-9.24, 1.96) |
| Post-CCN period | 42.11 | (32.92, 51.30) | 31.46 | (23.87, 39.04) |
| CCN appointment x post-CCN period | -15.08 | (-30.09, -0.07) | 11.00 | (2.21, 19.79) |
| *Age category (reference <45 years)* |  |  |  |  |
| 45-54 years | -0.18 | (-5.14, 4.77) | -0.26 | (-2.74, 2.23) |
| 55-64 years | -0.59 | (-5.58, 4.40) | -0.74 | (-3.41, 1.93) |
| ≥65 years | 1.20 | (-4.63, 7.04) | 1.10 | (-1.83, 4.02) |
| *Sex (reference Male)* |  |  |  |  |
| Female | -2.63 | (-6.94, 1.68) | -1.76 | (-4.34, 0.82) |
| *Race (reference White)* |  |  |  |  |
| Black | 6.59 | (2.28, 10.91) | 4.67 | (1.02, 8.31) |
| Other/Unknown | 2.20 | (-2.51, 6.91) | 1.64 | (-1.49, 4.78) |
| *Ethnicity (reference Non-Hispanic/Latino)* |  |  |  |  |
| Hispanic/Latino | 6.43 | (1.38, 11.48) | -6.68 | (-10.45, -2.91) |
| *Marital status (reference Married)* |  |  |  |  |
| Single/Other | 3.15 | (0.74, 5.55) | 0.93 | (-0.30, 2.16) |
| *VA copayment category (reference No copay)* |  |  |  |  |
| Some copay | 0.28 | (-2.87, 3.44) | 0.45 | (-1.04, 1.94) |
| Full copay | 2.20 | (-2.13, 6.53) | 4.04 | (1.32, 6.77) |
| New VA enrollee | -2.37 | (-7.56, 2.81) | -0.34 | (-2.86, 2.17) |
| *Insurance status (reference VA only)* |  |  |  |  |
| VA + private | 0.20 | (-4.79, 5.18) | -0.04 | (-2.41, 2.33) |
| VA + Medicare/Medicaid | 2.98 | (-0.90, 6.86) | 2.53 | (-0.50, 5.56) |
| VA, + private, + Medicare/Medicaid | 6.36 | (0.22, 12.50) | 5.85 | (2.79, 8.91) |
| Elixhauser score, 10 points | -0.83 | (-1.90, 0.24) | -0.71 | (-1.48, 0.05) |
| VA facility-level SHEP care coordination score | 0.48 | (0.13, 0.82) | 0.58 | (0.23, 0.93) |
| County-Level National ADI Rank | -0.09 | (-0.22, 0.05) | -0.12 | (-0.27, 0.03) |
| Mental health HPSA | -3.31 | (-7.46, 0.84) | -4.96 | (-10.62, 0.70) |
| *Rurality of residence (reference Urban)* |  |  |  |  |
| Rural | 1.42 | (-4.13, 6.97) | 0.26 | (-4.72, 5.24) |
| Highly rural | 2.49 | (-5.51, 10.49) | -1.24 | (-9.32, 6.84) |
| *Distance to nearest VA primary care facility (reference 0-5 miles)* |  |  |  |  |
| 6-10 miles | -2.81 | (-13.50, 7.87) | 0.89 | (-2.50, 4.27) |
| 11-20 miles | -0.30 | (-10.22, 9.63) | -1.43 | (-5.11, 2.25) |
| 21-40 miles | -0.1 | (-9.74, 9.54) | -4.13 | (-9.65, 1.40) |
| >40 miles | -1.76 | (-11.22, 7.70) | -2.68 | (-9.23, 3.87) |

Notes: Coefficients and 95% confidence intervals are from appointment-level multivariable linear regression models with VA facility-level cluster-robust standard errors.

Abbreviations: VA, Veterans Affairs; CCN, Community Care Networks; ADI, Area Deprivation Index; HPSA, Health Professional Shortage Area; SHEP, Survey of Healthcare Experiences of Patients.
